# Changes in Food Cravings and Eating Behavior after a Dietary Carbohydrate Restriction Intervention Trial

**DOI:** 10.3390/nu12010052

**Published:** 2019-12-24

**Authors:** Katherene O.-B. Anguah, Majid M. Syed-Abdul, Qiong Hu, Miriam Jacome-Sosa, Colette Heimowitz, Vicki Cox, Elizabeth J. Parks

**Affiliations:** 1 Department of Nutrition and Exercise Physiology, University of Missouri, Columbia, MO 65211, USA; ms9rf@mail.missouri.edu (M.M.S.-A.); huqiong313@gmail.com (Q.H.); parksej@missouri.edu (E.J.P.); 2 Department of Child Health, School of Medicine, University of Missouri, Columbia, MO 65212, USA; 3 Department of Internal Medicine, Division of Nutritional Science, Washington University School of Medicine, St. Louis, MO 63110, USA; mjacome@wustl.edu; 4 Atkins Nutritionals, Inc., Denver, CO 80202, USA; Cheimowitz@atkins.com; 5 Department of Nutrition, West Chester University, West Chester, PA 19383, USA; vickicuce@gmail.com

**Keywords:** carbohydrate restriction, food cravings, eating behaviors, weight loss

## Abstract

Compared to low-fat diets, low-carbohydrate (CHO) diets cause weight loss (WL) over a faster time frame; however, it is unknown how changes in food cravings and eating behavior contribute to this more rapid WL in the early phases of dieting. We hypothesized that reductions in food cravings and improved eating behaviors would be evident even after a relatively short (4-week) duration of CHO-restriction, and that these changes would be associated with WL. Adult participants (*n* = 19, 53% males, mean ± SD: BMI = 34.1 ± 0.8 kg/m^2^; age 40.6 ± 1.9 years) consumed a CHO-restricted diet (14% CHO, 58% fat, 28% protein) for 4 weeks. Before and after the intervention, specific and total cravings were measured with the Food Craving Inventory (FCI) and eating behaviors assessed with the Three-Factor Eating questionnaire. Food cravings were significantly reduced at week 4, while women had significantly greater reductions in sweet cravings than men. Dietary restraint was significantly increased by 102%, while disinhibiton and hunger scores were reduced (17% and 22%, respectively, *p* < 0.05). Changes in cravings were unrelated to changes in body weight except for the change in high-fat cravings where those who lost the most weight experienced the least reductions in fat cravings (*r* = −0.458, *p* = 0.049). Changes in dietary restraint were inversely related to several FCI subscales. A short-term, low-CHO diet was effective in reducing food cravings. These data suggest that in subjects that have successfully lost weight on a low-CHO diet, those who craved high-fat foods at the onset were able to satisfy their cravings—potentially due to the high-fat nature of this restricted diet.

## 1. Introduction

About 11% of the variance in eating behavior and weight gain can be explained by the experience of food cravings [1], which is generally defined as an intense desire to consume a particular kind of food that is difficult to resist [2,3]. Cravings differ from hunger in the intensity and specificity for the food craved for. Many overweight patients interested in weight loss (WL) may assume that dieting may increase their food cravings. Although a few WL studies suggest cravings may not change [4] or even increase [5,6], a large number of clinical trials, most from 12 weeks to 2 years in duration, have demonstrated that cravings are reduced during energy restriction [5,7,8,9,10,11,12,13,14,15,16,17,18]. Some authors have reported that greater WL is associated with greater reductions in food cravings [11,12,13], while others have found no relationships [5,19]. With regard to dietary macronutrient content, of the three studies investigating low-CHO diet effects on food cravings, two reported reduced cravings [11,15], while one [5] reported increased cravings. It is well recognized that low-CHO diets cause greater WL in the initial four weeks after adoption [20,21] and the duration of most past studies ranged from 12 weeks to two years. Only one study compared the effects of a low-fat and low-CHO diet on cravings for six weeks and found increased cravings on the low-CHO diet [6]. The tool or scale used to measure food cravings was not well defined in that paper and the sample size was small (n = 4). Thus, it is unknown whether food cravings change in the first four weeks of CHO restriction, a time when the WL trajectory is the steepest [20,21].

Very few studies have specifically investigated sex-related differences in cravings during WL. Anton and colleagues [9] found that compared to women, men reported stronger cravings for high-fat foods and fast-food fats at baseline and throughout the WL intervention, while women reported greater cravings for sweets and fruits/vegetables at baseline and throughout the intervention. Dorling et al. [22] also reported reductions in cravings for carbohydrates and fats at 24 months in males but not in females in a calorie-restricted group compared to an ad libitum intake group. Thus the little data available suggest sex-related differences in the types of foods craved for and this notion is supported by a 2016 review [23] that aimed to emphasize the relevance of investigating gender differences in food cravings.

Aside from food cravings, other eating behaviors affect WL success. The Three Factor Eating questionnaire (TFEQ) is a commonly used tool to assess eating behaviors termed dietary restraint, disinhibition, and hunger [24]. Higher baseline disinhibition was shown to be positively associated with baseline BMI and less WL success [25], while greater increases in dietary restraint were associated with greater WL [26,27,28] in obese subjects undergoing WL treatment. With regard to TFEQ hunger, in a recent study where a low-energy diet was fed for eight weeks, a one-unit higher hunger score at baseline and a one-unit reduction in scores of both disinhibition and hunger were associated with larger WL [29].

Given the effect of low-CHO diets to lead to relatively fast WL (within the first weeks of energy restriction), the purpose of the present analysis was to test the effect of a CHO-restricted diet (14% of energy) on self-reported food cravings and eating behaviors over four weeks. Furthermore, we also examined if there were any sex differences in food cravings and eating behavior. We hypothesized that with the exception of fruits/vegetables, all the FCI subscales would be reduced and that reductions in cravings would be correlated with WL, eating behaviors, and changes in blood glucose even during the early stages of WL. Furthermore, we hypothesized that CHO-restriction would result in increased restraint and decreased disinbition and hunger. The data on cravings and eating behaviors, analyzed here, were collected as part of a larger study designed to test the effect of the WL intervention on cardiovascular risk factors associated with metabolic syndrome. The results of that study have been previously published [30].

## 2. Materials and Methods

### 2.1. Study Participants and Design

Detailed descriptions of the participants and the study design for the parent study have been published previously [30]. The goal of the parent study was to determine whether four weeks of dietary CHO-restriction would reduce vascular stiffness assessed by aortic pulse wave velocity. The present paper focusses on results obtained from measures of cravings and eating behaviors assessed during this trial. The overall study was approved by the University of Missouri Health Science Institutional Review Board (Protocol #2004733) and written informed consent was obtained from all participants. This trial was registered on ClinicalTrials.gov Identifier: NCT00427193. Men and premenopausal women were recruited who were overweight or obese (BMI: 27–40 kg/m^2^), aged 30–55 years, and had characteristics of insulin resistance [31] or the metabolic syndrome [30]. With regard to physical activity, subjects had low to medium levels (5000 to 10,000 steps/day). Exclusion criteria included having diabetes, alcohol use of more than 5 standard drinks/week (70 g) for women and more than 10 standard drinks/week (140 g) for men, use of tobacco products, taking prescription medication for clinically significant endocrine, gastrointestinal, cardiovascular, hematological, hepatic, renal, respiratory, or genitourinary abnormalities or diseases, or being on any dietary regimen that would hinder adherence to the low-CHO diet.

**Figure 1:**
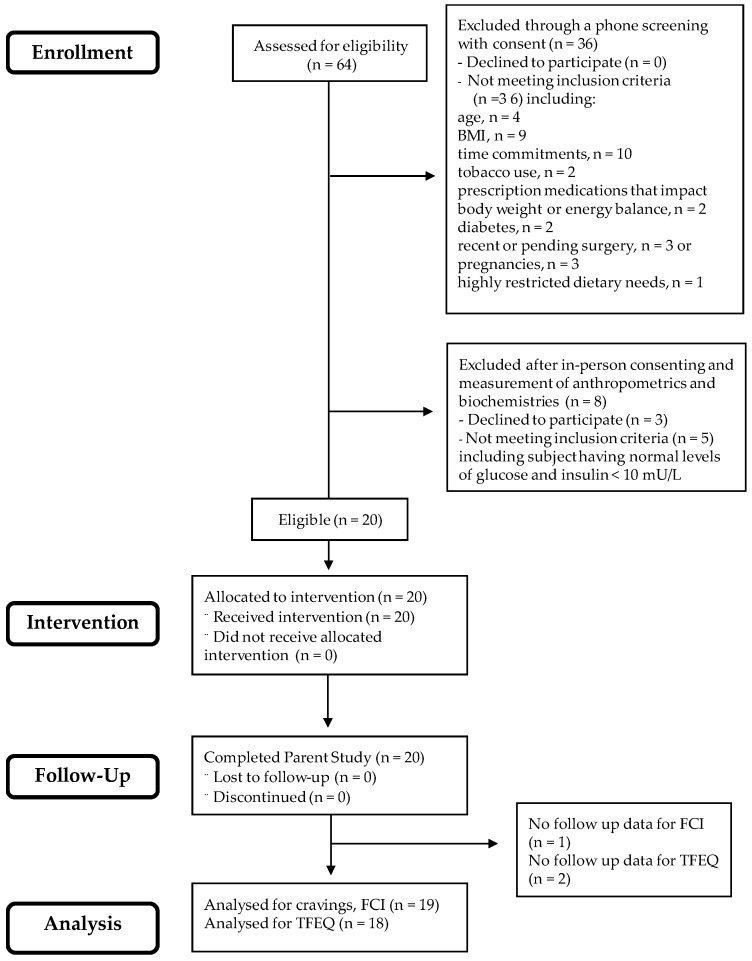
CONSORT diagram indicating the sample size at each stage of the study. This diagram is modified with permission from Syed-Majid et al. [30].

As shown in Figure 1, 64 participants were assessed for eligibility and, as per the original study design’s plan, 20 were assigned the CHO-restricted intervention [30]. To compare cravings with eating behaviors, WL, and biochemical outcomes (insulin and glucose concentrations), complete data were available for 19 of the original 20 subjects for food cravings and 18 out of the original 20 subjects for eating behaviors, TFEQ. The low-CHO diet provided 1500 kcal/day of energy and included 20–25 g of net CHO/day [30]. Subjects were provided all food for the first two weeks of the study, during which time they received comprehensive education to be able to cook and prepare their own low-CHO meals during weeks 3 and 4. Thus, for weeks 3 and 4, subjects consumed a diet similar to that eaten during the first two weeks by following the guidelines and using the educational materials given them by study investigators. Study staff were in contact with research participants on a near-daily basis to monitor dietary intake and assess food acceptance. Dietary adherence was assessed by inspection of returned food containers (first two weeks), respiratory quotient, and measurement of plasma ketones during week 4 [30]. Body weight was measured to the nearest 0.1 kg and fasting glucose and insulin concentrations were measured by enzymatic assay and ELISA, respectively [30].

### 2.2. Food Cravings

The Food Craving Inventory (FCI), originally developed by White et al. [32], has been previously validated and demonstrates internal consistency (Cronbach’s α = 0.76–0.93). The FCI is a 33-item questionnaire to assess cravings across five subscales: (1) high-fat foods (e.g., fried chicken, gravy, sausages, hot dogs), (2) sweets (e.g., cake, cinnamon rolls, ice cream, cookies), (3) CHO/starches (e.g., sandwich bread, rice, biscuits, pasta, pancakes), (4) fast-food fats (pizza, french fries, hamburger, chips), and (5) fruit/vegetable subscale (raw fruits, raw vegetables, canned fruit, fruit juice, and cooked vegetables). The fruit/vegetable subscale was not included in the original FCI by White and colleagues but has been previously used by others [9,13]. Items on the questionnaire are scored on a 1–5 scale with 1 = never and 5 = always/almost every day. A total score for each subscale is calculated by taking the average of scores for individual foods within that subscale. A total craving score is calculated from the aggregate or mean of all five subscales. For the present analysis, internal consistency (Cronbach’s α) was found for baseline and post low-CHO diet, respectively, for the high-fat food subscale (0.85 and 0.83), sweets (0.87 and 0.66), CHO/starches (0.88 and 0.80), fast-food fats (0.80 and 0.81), fruits/vegetables (0.80 and 0.76), and overall cravings (0.94 and 0.92). These values represent acceptable to good internal consistency of the scales used to measure food cravings.

### 2.3. Eating Behavior

The Three Factor Eating questionnaire (TFEQ), originally developed by Stunkard and Messick [24], is a 51-item questionnaire with three subscales: a 21-item dietary restraint scale, a 16-item dietary disinhibition scale, and a 14-item hunger scale. The dietary restraint scale measures cognitive control of eating, i.e., the tendency to consciously restrict or control food intake as a means of controlling weight, while dietary disinhibition scale measures loss of cognitive control of eating which is the tendency to overeat in response to negative emotional states or the presence of highly palatable foods, and the hunger scale measures susceptibility to feelings of hunger.

### 2.4. Statistical Analyses

All statistical analyses were performed by using the Statistical Package for Social Sciences (IBM SPSS Statistics, version 25, SPSS Inc., Armonk, NY, USA). Participant characteristics are expressed as mean ± SD, while mean cravings, and eating behavior scores (dietary restraint, dietary disinhibition, and hunger) are expressed as mean ± SE. Statistical significance was accepted at *p* < 0.05. For the food cravings data, analyses focused on each participant’s aggregated cravings ratings, which were computed by averaging an outcome rating for foods in the same category (high fat, sweet, CHO/starches, fast-food fats, fruits/vegetables) for baseline, and week 4 (post low-CHO diet). For each of the 33 items measuring food cravings on the FCI, a total craving score was calculated as the average of the overall score for high fat, sweet, CHO/starches, fast-food fats, and fruits/vegetables. Thus, six craving variables (high fat, sweets, CHO/starches, fast-food fats, fruits/vegetables, and total cravings) were included as outcomes. Similarly, for the eating behaviors, an aggregated score for each of three constructs (dietary restraint, disinhibition, and hunger) was calculated by averaging the scores obtained by each subject for each of the three different constructs. A paired-sample t-test was used to assess differences in mean cravings scores, dietary restraint, dietary disinhibition, and hunger between baseline and post low-CHO diet (week 4).

A repeated-measures ANOVA with sex as the between-subject factor and time (baseline, post low-CHO diet) as the within-subject factor was carried out to test the main effect of sex on cravings and eating behaviors. Pearson correlations were used to assess the relationships between change in cravings and change in body weight, change in eating behavior, and change in insulin and glucose concentrations. Change variables were calculated by subtracting the value at baseline from the value at week 4. Thus, a positive value indicates increased cravings, restraint, disinhibition, hunger, weight gain, glucose and insulin concentrations, while a negative value implies decreased cravings, restraint, disinhibition, hunger, weight loss, glucose and insulin concentrations. Percent changes were calculated by dividing the difference between baseline and week 4 values by the baseline value and then multiplying the answer by 100 for each subject. The average was then taken as the percent change for the variable in question. This approach allowed us to capture variability in responses within individual subjects.

## 3. Results

### 3.1. Participants, Biochemical Indices and Body Weight

**Figure 2:**
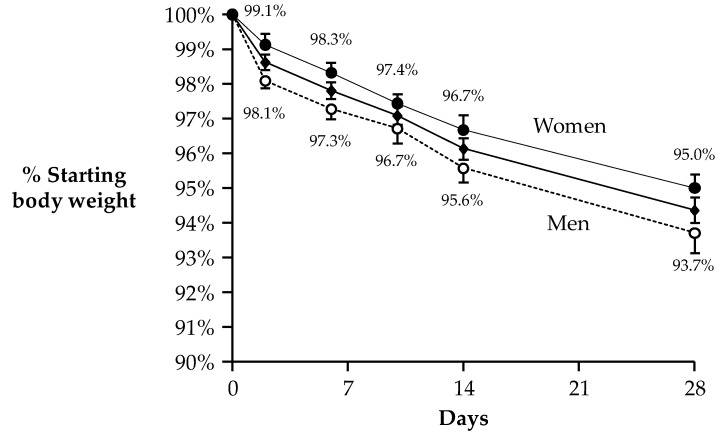
Body weight loss (WL) over 4 weeks of a low-carbohydrate (low-CHO), energy restricted diet. Data are mean ± SE and reported for N = 19 subjects who had complete data for food cravings. Analysis of body WL over time revealed significant effects for men and women combined (center line, with diamond symbols) and also separately for the sexes. WL in men (dotted line with open circles) was significantly greater than in women (top line with closed circles, *p* = 0.002, ANOVA effect of sex). This figure is modified from Syed-Majid et al. [30] with permission.

Baseline characteristics are presented in Table 1; subjects were middle-aged and had BMIs in the overweight to obese range. Fasting glucose concentrations were normal at baseline and did not change after WL (95.5 ± 2.2 vs. 90.3 ± 3.0 mg/dL, *p* = 0.139). Baseline fasting insulin concentrations tended to be elevated (12.2 ± 1.2 mU/L) and fell 22% with WL (8.8 ± 0.6 mU/L, *p* = 0.003). Participants lost 6.0 ± 0.5 kg from baseline to week 4 representing ~6% of their baseline body weight. In men compared to women, a total of 7.2 ± 0.7 kg and 4.7 ± 0.5 kg was lost from baseline to week 4, representing 6.3% and 5.0% of baseline body weight, respectively (treatment × sex effect, *p* = 0.011). Men lost significantly more weight than women (*p* = 0.002, ANOVA effect of sex). However, both men and women lost a significant amount of weight from baseline. Weight loss trajectories for all subjects, and men and women are shown in Figure 2. All participants reported that they were either somewhat satisfied (45%) or extremely satisfied (55%) with the diet post-intervention (data not shown).

### 3.2. Food Cravings and Eating Behaviors

**Figure 3:**
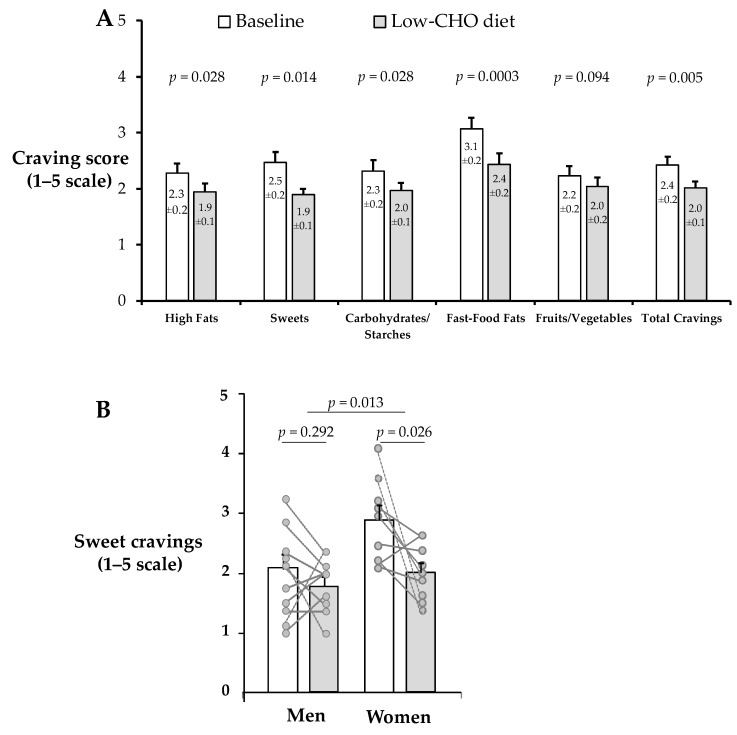
Mean craving scores over time (**A**), and sweet cravings by sex (**B**). Data are mean ± SE and reported for N = 19 subjects (10 men and 9 women) who had complete cravings data. A score of 1 = never and 5 = always/almost every day. All cravings subcategories except fruits/vegetables were significantly reduced in the total sample. Only sweet cravings showed a significant main effect of sex. All significance tests were two-tailed.

As shown in Figure 3A, cravings were significantly reduced from baseline to week 4 (post low-CHO diet) for most cravings subscales: high fat (−0.3 ± 0.1, *p* = 0.028), sweets (−0.6 ± 0.2, *p* = 0.014), CHO/starches (−0.4 ± 0.1 , *p* = 0.028), fast-food fats (−0.6 ± 0.1, *p* = 0.0003), and total cravings (−0.4 ± 0.1, *p* = 0.005). By contrast, cravings for fruits/vegetables only tended to be reduced (−0.2 ± 0.1, *p* = 0.094). Overall, for the majority of reductions in cravings, women exhibited greater effects than men, although only for sweet cravings— this effect significant *(p* = 0.013). As shown in Figure 3B, sweet cravings dropped for all but one woman whereas in men, changes in sweet cravings were variable.

**Figure 4:**
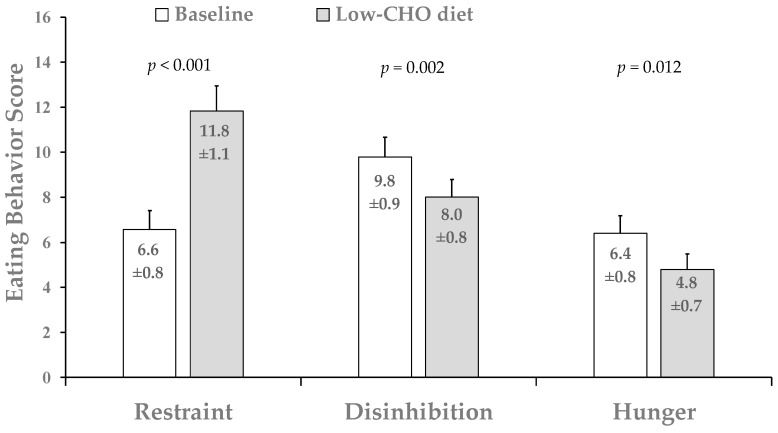
Mean eating behavior scores in the total sample. Data are mean ± SE and reported for N = 18 subjects (9 men and 9 women with complete data for TFEQ). All significance tests were two-tailed.

Dietary restraint was significantly increased (~102 ± 21%) from baseline to week 4 after the low-CHO diet, while both disinhibition (~17 ± 7%) and hunger scores were significantly reduced (22 ± 9%) from baseline to week 4 in the entire cohort (Figure 4). No significant main effect of sex was found for any of the three TEFQ subscales (*p* > 0.05).

### 3.3. Food Cravings Correlations with Body Weight, Glucose and Eating Behavior

**Figure 5:**
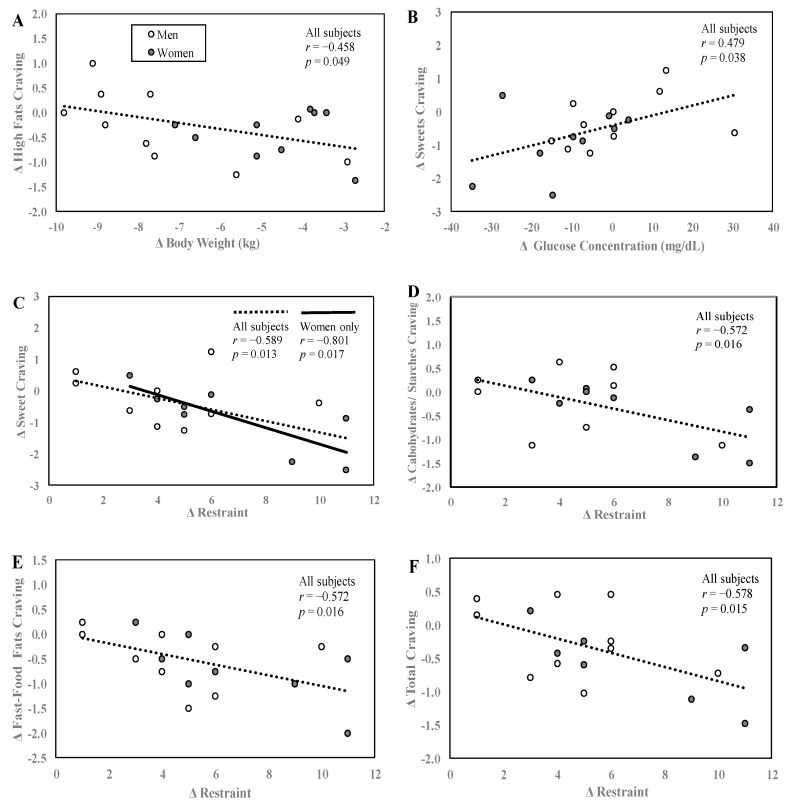
Relationships between craving scores, body weight, glucose concentrations, and eating behaviors. Correlations between change in high-fat cravings and change in body weight (**A**), change in sweet cravings and change in blood glucose concentration (**B**), and change in sweet, CHO/starches, fast-food fats, total cravings and dietary restraint (**C**–**F**). Change values were calculated by subtracting the value at baseline from week 4. Data are reported for N = 19 subjects that had complete data for cravings, body weight and glucose concentration (**A**,**B**) and N = 17 subjects who had complete data for both cravings and TFEQ (**C**,**D**). All significance tests were two-tailed.

At baseline, within the FCI, the higher the baseline sweet cravings scores, the greater sweet cravings fell during the intervention (r = −0.865, *p* < 0.0001, data not shown). The same was true for high fat cravings (r = −0.566, *p* = 0.012). At baseline, between food craving scores and eating behaviors scores, no significant relationships were found. With regard to changes in these variables over time, Figure 5A–F shows the relationships between changes in food cravings and eating behaviors, body weight, and plasma glucose concentrations. Changes in cravings for high-fat foods from baseline to week 4 were significantly negatively related to change in body weight (Figure 5A). In other words, although all subjects lost weight during the WL intervention, subjects who lost the most weight experienced the smallest reductions in cravings for high-fat foods. This relationship was lost when controlled for by baseline high-fat craving levels, which suggest that the starting level of high-fat craving governed the change. No other significant correlations were observed for changes in the other craving variables and changes in body weight. Changes in sweet cravings were significantly positively related to the changes in blood glucose concentrations (Figure 5B) such that those who exhibited the greatest reductions in blood glucose had the greatest reductions in cravings for sweets. No other significant correlations were observed between changes in glucose and changes in the other craving subscales. Changes in cognitive restraint were also statistically negatively correlated with changes in sweet cravings (Figure 5C). A similar significant negative trend was observed for changes in cognitive restraint and three other cravings subscales: changes in CHO/starch cravings (Figure 5D), changes in fast-food fat cravings (Figure 5E) and changes in overall cravings (Figure 5F). Thus, those individuals whose cravings for sweets, CHO/starches, fast-food fats, and total cravings decreased the most also had the greatest increases in cognitive restraint.

## 4. Discussion

In this study, we investigated changes in cravings and eating behaviors during a 4-week low-CHO, WL intervention to understand how changes in cravings were related to eating behaviors and some biochemical indices. We also explored sex differences in cravings and eating behaviors following the intervention. We hypothesized that with the exception of fruits/vegetables, all the FCI subscales would be reduced and that reductions in cravings would be correlated with WL, eating behaviors, and blood glucose. Furthermore, we hypothesized that CHO-restriction would result in increased restraint and decreased disinbition and hunger. First, consistent with previous studies of longer duration [5,7,8,9,10,11,12,13,14,15,16,17], our data showed a reduction in specific and total cravings after only 4 weeks. In an elegant study by Kahathuduwa et al. [18], a low-energy diet fed for 3 weeks resulted in similar WL (~4 kg) and significant reductions in cravings for sweets and starchy foods. These lower cravings were supported by fMRI data showing decreased activations in brain reward regions—whether similar fMRI changes would be observed with a low-CHO diet is not known. Our findings that most food cravings subscales and total cravings were reduced support the classical conditioning model [33] for cravings and not the deficiency model [2,34,35]. If the theory of deficiency were to hold, then we would expect cravings to increase with the low-CHO, WL diet.

Second, no sex specific effects on cravings were observed, except for changes in sweet cravings where women exhibited greater reductions in cravings than men. Although this line of research has not been extensively studied, according to a 2016 review of cravings in obese individuals [23], sex differences in cravings were found for (1) the types of food craved (women tended to crave sweets while men craved savory foods), (2) the frequency and intensity of food craved (women reported higher craving scores and frequency that men, and (3) the regulation of food craving (women reported it was harder to regulate food cravings than men). Women are disproportionately burdened by overweight/obesity and that previous data show that women may be less succesful at regulating cravings and report higher cravings for sweets, compared to men [23]. Our finding that women were more successful at reducing sweet cravings is encouraging and supports the idea that women may have benefitted more from the short-term CHO-restriction than men. Our data suggest that sex differences in sweet cravings should be taken into consideration when designing dietary regimens for weight loss.

Third, dietary restraint was increased in the total sample—a finding that is consistent with previous reports [22,36], while disinhibition and hunger were reduced. Furthermore, our correlational analyses demonstrated that changes in cravings were mostly unrelated to changes in body weight. The one exception was that those who lost the most weight exhibited the smallest reductions in cravings for high-fat foods; this relationship was lost after controlling for baseline high-fat. One interpretaton of this result might be that those individuals who preferred high-fat foods at baseline, had their food preference met by the low-CHO (high-fat) diet, which supported dietary adherence, leading to more body WL. Thus, alignment of a subject’s cravings with the macronutrient content of the diet could contribute to additional WL success. How their sustained cravings for high-fat foods was related to this scenario is not known. Gilhooly et al. [4] found that over a 6-month WL intervention of low-glycemic and high-glycemic load foods, subjects who lost a greater percentage of weight also craved higher energy dense foods at month 6 but also reported giving in to food cravings less frequently. This supports the notion that, it is the behavior that follows food cravings (resisting food cravings or feeling in control of eating) rather than the intensity or frequency of cravings that contributes to successful WL [37]. The fact that the significant relationship between change in high-fat craving and change in body weight was lost after controlling for baseline fast-food craving suggests that the initial level of high-fat cravings drove the change. Also, the greater the reductions in cravings for sweets, CHO/starches, fast-food fats, and total cravings, the greater the dietary restraint during the WL intervention. These observations suggest that cravings and dietary restraint are related to each other. Lastly, those individuals whose cravings for sweets were reduced the most experienced the greatest reductions in blood glucose concentrations, which suggests that the connection between these two events may be mediated by reductions in dietary CHO intake. 

Strengths of this study include the controlled dietary intake with the provision of some of the foods. This may have made it easier for subjects to adhere to the diet and allowed the investigation of changes in food cravings and eating behaviors under controlled conditions. The use of validated questionnaires to assess food cravings and eating behaviors increased rigor, as did the use of biological measures to test dietary adherence (blood ketones, indirect calorimetry [30]). Objectively measuring compliance to WL diets is paramount to explaining weight changes as some have argued that it is the adherence to the dietary regimen rather than changes in variables such as cravings and eating behavior that moderates weight loss [16,38,39].

Study limitations included the lack of a control group not on a low-CHO diet; the current results warrant a larger study of randomized design. The use of a standardized diet for all participants may have resulted in men losing more weight than women and the sample size was small, which is a common limitation of studies where food intake is highly controlled. Nonetheless, the number of subjects was sufficient to statistically detect within-subject-differences. It is possible that the study was not sufficiently powered to be able to detect relationships between outcomes of interest by correlational analyses and hence the inability to see significant relationships between changes in cravings and changes in body weight for most of our craving subscales. Given an α of 0.05, power of 80%, and effect size of Pearson’s r of 0.5 (large) as proposed by Cohen [40], the sample size required for statistically significant correlations was 29 assuming a null hypothesis of zero correlation. In our correlational analysis, we had complete data for 17–19 subjects depending on the outcome of interest.

## 5. Conclusions

In summary, a low-CHO diet promoted significant WL over a short time-frame and resulted in significant reductions in specific and total cravings, reductions in disinhibition and hunger, and increased dietary restraint in the total sample studied. Greater increases in dietary restraint resulted in greater decreases in some specific and total cravings. Women exhibited greater reductions in sweet cravings than men and thus, dietary strategies aimed at ameliorating sweet food cravings may need to take sex into consideration. Though all participants lost weight, our data showed that a greater WL induced by a low-CHO diet was associated with smaller reductions in high-fat cravings but was not significantly related to any other specific or total craving. This relationship was lost when controlling for baseline levels for high-fat food cravings which suggests that when high-fat cravings are elevated before WL, a low-CHO diet may be efficacious. Due to the preliminary nature of our findings, future studies should be conducted using a randomized, controlled design with a larger sample size and also control for sex differences in energy requirements. These data support the conditioning model [33] for cravings and the relationship between high fat cravings and body weight reductions suggests that subjects that crave high-fat foods may be able to satisfy their cravings with a low-CHO, high-fat diet and, thus, improve their WL success.

## Figures and Tables

**Table 1:** Baseline characteristics of study participants ^1^.

Characteristic	All (*n* = 19)	Men (*n* = 10)	Women (*n* = 9)
Age (year)	40.6 ± 8.4	41.8 ± 10.2	39.2 ± 6.3
Body weight (kg)	103.4 ± 16.9	113.8 ± 15.4	91.9 ± 9.6
Height (m)	1.74 ± 0.10	1.82 ± 0.06	1.65 ± 0.03
BMI (kg/m^2^)	34.1 ± 3.3	34.2 ± 3.0	34.0 ± 3.8
Plasma insulin (mU/L)	12.2 ± 5.2	11.6 ± 3.9	12.8 ± 6.6
Plasma glucose (mg/dL)	95.5 ± 9.6	93.5 ± 7.9	97.8 ± 11.3
Physical activity (steps)	7295 ± 2549	8586 ± 2897	5860 ± 887

^1^ Data are mean ± standard deviation.
